# Genomics of Fibromuscular Dysplasia

**DOI:** 10.3390/ijms19051526

**Published:** 2018-05-21

**Authors:** Silvia Di Monaco, Adrien Georges, Jean-Philippe Lengelé, Miikka Vikkula, Alexandre Persu

**Affiliations:** 1 Division of Cardiology, Cliniques Universitaires Saint-Luc, Université Catholique de Louvain, 1200 Brussels, Belgium; silvia.dimonaco@gmail.com (S.D.M.); Jean_Philippe.LENGELE@ghdc.be (J.-P.L.); 2 Department of Medical Sciences, Internal Medicine and Hypertension Division, AOU Città della Salute e della Scienza, University of Turin, 10124 Turin, Italy; 3 INSERM, UMR970 Paris Cardiovascular Research Center (PARCC), F-75015 Paris, France; adrien.georges@inserm.fr; 4 Department of Nephrology, Grand Hôpital De Charleroi, 6060 Gilly, Belgium; 5 Human Molecular Genetics, de Duve Institute, Université catholique de Louvain, 1200 Brussels, Belgium; miikka.vikkula@uclouvain.be; 6 Pole of Cardiovascular Research, Institut de Recherche Expérimentale et Clinique, Université catholique de Louvain, 1200 Brussels, Belgium

**Keywords:** fibromuscular dysplasia, non atherosclerotic vascular stenosis, *PHACTR1*, genetic association, cervical artery dissection, spontaneous coronary arteries dissection

## Abstract

Fibromuscular Dysplasia (FMD) is “an idiopathic, segmental, non-atherosclerotic and non-inflammatory disease of the musculature of arterial walls, leading to stenosis of small and medium-sized arteries” (Persu, et al; 2014). FMD can lead to hypertension, arterial dissections, subarachnoid haemorrhage, stroke or mesenteric ischemia. The pathophysiology of the disease remains elusive. While familial cases are rare (<5%) in contemporary FMD registries, there is evidence in favour of the existence of multiple genetic factors involved in this vascular disease. Recent collaborative efforts allowed the identification of a first genetic locus associated with FMD. This intronic variant located in the phosphatase and actin regulator 1 gene (*PHACTR1*) may influence the transcription activity of the endothelin-1 gene (*EDN1*) located nearby on chromosome 6. Interestingly, the *PHACTR1* locus has also been involved in vascular hypertrophy in normal subjects, carotid dissection, migraine and coronary artery disease. National and international registries of FMD patients, with deep and harmonised phenotypic and genetic characterisation, are expected to be instrumental to improve our understanding of the genetic basis and pathophysiology of this intriguing vascular disease.

## 1. Definition and Main Features of Fibromuscular Dysplasia (FMD)

Fibromuscular Dysplasia (FMD) has been defined as “an idiopathic, segmental, non-atherosclerotic and non-inflammatory disease of the musculature of arterial walls, leading to stenosis of small and medium-sized arteries” [1]. In the last years, the perception of FMD has evolved from a rare cause of secondary hypertension involving renal arteries to a more diffuse vascular disease, affecting also cervico-cephalic, visceral, coronary and iliac arteries [1,2].

Renal and cervico-cephalic arteries are the most frequently affected vascular beds, and both lesions often coexist (65% in the US registry) [3]. In the Assessment of Renal and Cervical Artery Dysplasia (ARCADIA) Belgian-French registry [4], systematic, state of the art exploration of the main arterial beds in 469 patients with FMD revealed that 48% of patients have focal or multifocal FMD lesions of two vascular beds or more and 15% of three vascular beds or more (multivessel FMD). According to the affected arterial bed, FMD can lead to hypertension (renal artery FMD), as well as to severe complications including arterial aneurysms and dissections, subarachnoid haemorrhage, stroke or mesenteric ischemia [1,5]. While FMD was initially considered as a rare disease, silent FMD lesions have been detected in 3–6% of potential kidney donors [6,7].

Recently, multifocal FMD lesions of extracoronary vascular beds have been also identified in up to 75–80% of patients with spontaneous coronary artery dissection (SCAD). Patients who have had a SCAD share many similarities with FMD patients, including female predominance (>90%), age at diagnosis (≈50 years old) and few cardiovascular risk factors [8]. FMD affects predominantly women: 91% in the US registry [3], 84% in the ARCADIA study [4], and 83% in the European registry (A. Persu, personal communication). In contemporary cohorts, the age at diagnosis is in the range of 50–55 years, with a wide distribution from small infants [9] to octogenarians [4,5].

Based on angiographic patterns two subtypes have been defined for renal FMD [1,5,10], and subsequently for cervico-cephalic FMD [1,11], i.e., (i) multifocal FMD: “string-of-beads” appearance, alternation of stenosis and aneurysmal dilations (>80% of cases), corresponding to the medial form according to the former histological classification [12]; and (ii) focal FMD: isolated stenosis, whatever its length, in young patients (usually <40 year old) with few or no cardiovascular risk factors, in the absence of atherosclerotic or inflammatory lesions. While the pattern of multifocal FMD is almost pathognomonic, in order to establish the diagnosis of focal FMD, it is necessary to rule out early localized atherosclerosis, as well as a series of inflammatory and genetic arteriopathies (see Table 1).

Until recently, the aetiology of FMD remains elusive. The classical aetiological hypothesis are summarized in the next section.

## 2. Classical Aetiological Hypothesis

### 2.1. Female Hormones

In view of the predominance of women among FMD patients, a role for female hormones has been assumed. In particular, it has been hypothesized that the estrogen-induced production of extracellular matrix proteins from vascular fibroblasts and vascular smooth muscle cells [13], may be responsible for some typical histopathological alterations of FMD [12,14]. Unfortunately, up to now, we lack consistent and powerful data to assess for associations between FMD and e.g., the number of pregnancies, age at menarche, history of hysterectomy, gynaecological problems, spontaneous abortion and/or oral contraceptive therapy [12,15]. In addition, all available studies are retrospective, small and underpowered. Therefore, large prospective registries are needed to test the female hormonal environment hypothesis.

### 2.2. Traumatic Theory

Other triggers of FMD may include arterial wall stress [12,14], as it was observed that cyclic vascular wall stretch increased synthesis of matrix components by smooth muscle cells [16,17]. It has also been hypothesized that FMD might result from renal artery traction due to nephroptosis [15] or repetitive micro-traumas of the vascular wall induced by excessive arterial pulsatility [18]. However, longitudinal studies that would allow proving or disproving these hypotheses are lacking.

### 2.3. Intramural Ischemia

In 1970s, it was proposed that the first determinant of FMD lesions is occlusion of the vasa vasorum, which may induce hypoxia in the arterial wall and transformation of smooth muscle cells into myofibroblasts, typically found in histopathological samples of arteries affected by FMD [19,20]. In particular, Sottiurai and co-workers demonstrated an increase in myofibroblasts and extra-cellular connective tissue of the media, as a result of vasa vasorum obliteration by injection of a thrombine–gelatine mixture into dog femoral arteries [19]. Furthermore, the extracranial internal carotid and external iliac arteries have fewer vasa vasorum than other muscular arteries of similar size, which may make them more susceptible to intramural ischemia [12,15]. However, occlusion of vasa vasorum in patients with FMD has not been demonstrated [20].

### 2.4. Smoking

Since 1979, smoking has been associated with genesis of FMD and/or its progression in at least 6 studies [15,21,22,23,24,25]. In particular, in a French cohort including 337 patients with FMD, the proportion of current and ever smokers was 30% and 50%, respectively, compared to 18% and 37% in 337 essential hypertensive controls (*p* ≤ 0.001) [24]. Furthermore, current smoking compared to non-current smoking was associated with an earlier FMD onset (43 vs. 51 years, *p* < 0.001) [24], and a higher proportion of renal asymmetry (21% vs. 4%, *p* = 0.001) and number of renal artery revascularization interventions (57% vs. 31%, *p* ≤ 0.001) [24]. Finally, in the US Registry, ever smokers were characterized by an increased proportion of aortic (4.8% vs. 1.5%, *p* < 0.01) [26] but also intracerebral (4.8% vs. 1.7%, *p* < 0.01) aneurysms [25] compared to never smokers.

## 3. FMD as a Genetic Disease

### 3.1. Familial Forms of FMD

**Figure 1:**
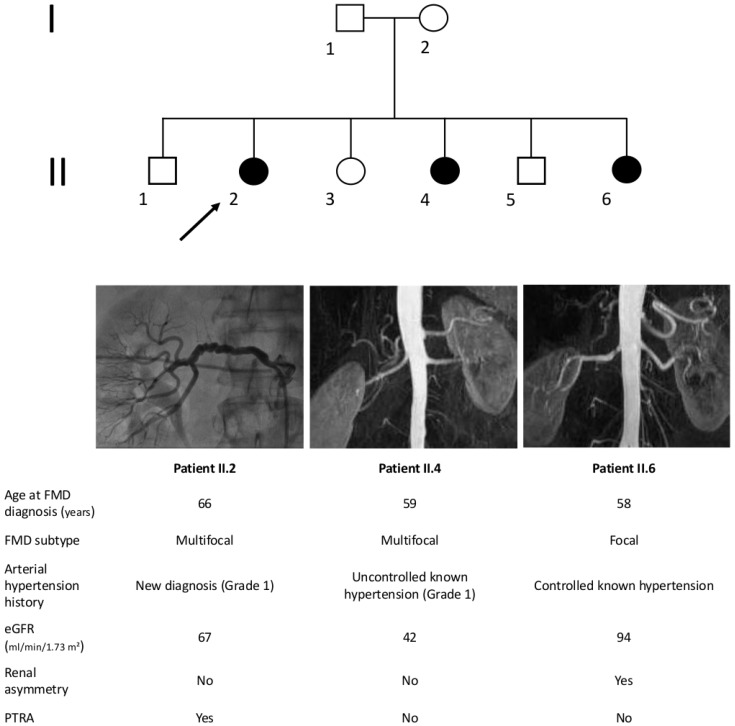
Example of familial FMD from Belgian Multicentric FMD Cohort (BEL-FMD). Patient II.2 came to clinical attention at the age of 64 years for de novo arterial hypertension. The abdominal Computed Tomography Angiogram showed an aspect compatible with multifocal FMD of both renal arteries, significant on the right side. She underwent right percutaneous transluminal renal angioplasty (PTRA), and the hypertensive crises regressed. Patient II.4 was hypertensive from the age of 53 years and was referred at the age of 59 years for worsening of blood pressure control, associated to decreased renal function. Abdominal Magnetic Resonance Angiography (MRA) showed mild irregularities suggestive of FMD in the right renal artery, in the absence of significant stenosis. Patient II.6 came to clinical attention at the age of 58 years for renal asymmetry (kidney length: 9.5 cm on the right side vs. 12 cm on the left). The abdominal MRA identified a focal stenosis of 70% of the right renal artery. Patient II.6 underwent renal arteriography, which disclosed an irregular aspect of the arterial wall on the left side and only a 30% stenosis on the right side. Notably, none of these three patients had lesions of cervico-cephalic or others vascular beds. Estimated Glomerular Filtration Rate (eGFR) was calculated using the Modification of Diet in Renal Disease (MDRD) equation.

Since 1960s to 1970s, a number of cases of familial FMD, i.e., occurrence of FMD in two or more siblings or relatives, have been reported [26,27,28,29,30,31]. We report a case of 3 siblings affected by hypertension and renal artery FMD followed at the Grand Hôpital de Charleroi, Gilly, Belgium (Figure 1), as an illustration. Interestingly, while two of them had typical string-of-beads lesions, the third had an isolated stenosis compatible with focal FMD.

In the absence of systematic exploration of first-degree relatives of patients with FMD, the prevalence of familial FMD remains unclear. In a French cohort of 100 unrelated patients with renal artery FMD, FMD lesions were documented by angiography in at least one first-degree relative in 11% of patients [32]. In the first report of the US registry, 7.3% of 447 patients reported a history of FMD in at least another 1st or 2nd degree relative [3]. However, in recent reports this percentage tends to be substantially lower: 2.9% in the ARCADIA study [4] and 2.8% in European registry (A. Persu, personal communication). The prevalence of familial FMD may be higher in paediatric than in adult patients (17.2% of 29 children vs. 4.7% of 864 adult patients with FMD in a recent report of the US registry) [33].

### 3.2. Genetic Susceptibility to FMD

While clear Mendelian familial presentation of FMD appears to be rare, genetic factors may be involved in the pathophysiology of the more common, apparently sporadically occurring forms of FMD. Already in 1980, Rushton suggested a familial pattern of FMD in 12 out of 20 unrelated FMD patients, with indication of autosomal dominant inheritance [34]. Nevertheless, the presence of FMD in relatives was assumed on the basis a history of vascular diseases occurring before 50 years, many of which may have been due to atherosclerosis [34].

More than 20 years later, using high resolution echotracking, Boutouyrie and co-workers described typical abnormalities of the arterial wall of the carotid bifurcation and radial artery of patients with renal artery FMD, including a characteristic “triple signal” pattern [23]. Interestingly, these abnormalities were significantly more frequent in patients with FMD (echotracking arterial score 4.02) but also in apparently healthy first-degree relatives of patients with FMD (echotracking arterial score 4.17) compared to unrelated normotensive controls (echotracking arterial score 2.52), with a pattern of inheritance suggesting once again an autosomal-dominant transmission [35]. Beyond the existence of a small proportion of clearly familial forms, these data suggest the existence of an inherited component in FMD at large, and provide a rationale for recent studies aiming at identifying susceptibility genes.

### 3.3. Overlap with Genetic Syndromes

Vascular abnormalities associated with FMD include arterial stenosis, tortuosity, aneurysms and dissections in one or more arterial beds [1]. Patients with rare inherited arteriopathies and connective tissue diseases (CTD) may harbour similar lesions. Furthermore, a proportion of patients with FMD harbour non-vascular features associated with CTD, suggesting an overlap between these entities and a possible role of the corresponding genes in non-syndromic FMD. In a cohort of 47 multifocal FMD patients, without family history of CTD, 95.7% of subjects presented radiological features, such as cerebral aneurysm (12.8%), early onset degenerative spine disease (95.7%), increased incidence of Arnold-Chiari I malformation (6.4%) and dural ectasia (42.6%) [36]. However, genetic screening failed to identify mutations in genes involved in hereditary CTD (*COL3A1*, *FBN1*, *PLOD1*, *TGFβR1*, *TGFβR2*, *TGFβ2*, *SMAD3*, *ACTA2*, and *COL5A1*) [36]. The most frequent clinical CTD features in a cohort of 139 female patients with FMD from the US registry were early onset (before age 50) osteoarthritis (15.6%), palatal abnormalities (56.1%), moderately severe myopia (29.1%), pectus excavatum or carinatum (7.2%), and dental crowding (29.7%) [37]. However, the prevalence of classical CTD features was estimated at 18.7%, similar to the general female population [37]. Finally, no mutation in *TGFβR2*, *COL3A1*, *FBN1*, *ACTA2*, or *SMAD3* gene was detected in a series of 35 FMD patients, again from the US registry. In this cohort, two distinct variants were identified in transforming growth factor beta receptor 1 (*TGFβR1*) gene in two unrelated patients. Both variants induced an amino acid substitution in a highly conserved region of *TGFβR1*, however their role is unknown [38]. Still, these findings may be relevant in view of the identification of increased plasma transforming growth factor beta-1 (TGF-β1) and transforming growth factor beta-2 (TGF-β2) secretion in dermal fibroblast lines from patients with FMD compared to age and gender-matched controls [36]. Overall, while FMD and CTD share some similarities, arguments in favour of a common genetic basis are scarce.

## 4. Challenges of the Genetic Investigation of FMD

Several reasons make genetic investigation of FMD challenging and limit access to multi-generational families or large cohorts of patients: (i) as most patients are no longer operated nowadays, histopathologic confirmation of the diagnosis is lacking; (ii) the surrogate “gold standard”, catheter-based angiography, which was at the basis of the current angiographic diagnostic criteria and classification cannot be proposed as a first-line screening test, in view of its invasiveness and potential risks [1]; (iii) while the string of beads is almost pathognomonic for multifocal FMD, the diagnosis of focal FMD requires exclusion of a number of FMD mimics, such as localized atherosclerosis, and inflammatory or inherited arteriopathies (Table 1), which implies a risk of misclassification and subsequent decrease in statistical power in case-control studies; (iv) in the absence of systematic exploration of controls and relatives of FMD patients, study results may be unreliable, as it has been estimated that 3–6% of subjects from the general population may harbour silent FMD lesion [6,7]; and (v) few large, multigenerational families are currently available for analysis. These hurdles may be partly overcome by the development of large national and international registries [3,4,39] including well characterized patients, with image archiving and biobanking, and the use of standardized, state-of-art imaging, as well as identification and validation of specific biomarkers, such as subtle changes in the arterial wall detected by high resolution echography [23,35,40,41].

## 5. Current Knowledge from Genetic Studies

### 5.1. Candidate Gene Studies

Literature on the genetics of FMD is scarce and, when available, studies are generally underpowered to assess the role of the tested genes. The main reason is the lack of large pedigrees to perform linkage analyses or large cohorts of well-characterized FMD patients for association studies. Variants in genes belonging to pathways related to the extracellular matrix of the arterial wall failed to show significant or even promising associations with FMD. Single Nucleotide Polymorphisms (SNPs) in the elastin gene (*ELN*), which harbours causative mutations for supravalvular aortic stenosis in Williams–Beuren syndrome (OMIM reference: #194050) (Perdu & Jeunemaitre, Unpublished data), as well as in the alpha1-antitrypsin gene (*AAT*) [42], were distributed evenly in patients with FMD and healthy controls or patients with essential hypertension. The modest sample size (*N* cases = 161) and lack of comprehensive coverage of the genes of interest, including regulatory regions, may partially explain these negative findings. Screening for causative mutations in the actin α-2 gene (*ACTA2*), involved in rare cases of aortic aneurysms was also negative [43]. Finally, as indicated above, screening for mutations involved in rare vascular syndromes such as Marfan, Loeys-Dietz and vascular Ehlers-Danlos Syndrome, in the TGFβ signalling pathway and/or extracellular matrix components (e.g., fibrillin and collagen genes) was disappointing [26,37]. Fortunately, the development of Next Generation Sequencing (NGS) opened the possibility to conduct hypothesis free experiments, such as exome sequencing, to explore families.

### 5.2. Exome Studies

#### 5.2.1. Genetic Investigation of FMD Using Exome Sequencing in Families

The first exome sequencing study published on FMD was conducted in 16 related patients from 7 families including at least two first-degree relatives with confirmed FMD recruited in the French database of the Rare Vascular Diseases Referral Centre of the European Hospital Georges Pompidou (HEGP), Paris, France [31]. Under the hypothesis of implication of rare variants, the authors applied a filter on minor allele frequency <0.01, and only analysed coding variants that changed amino acids. Affected siblings share 50% of their genetic variants by chance. However, if several genetic variants located in the same gene are shared by siblings from several families, one can argue for genetic causality for this gene. A threshold of three families sharing variants in the same gene was arbitrary chosen in order to consider a gene as a putative candidate for FMD. Unfortunately, this condition has not be satisfied by any of the 3971 genes analysed [31], suggesting the lack of major common genetic determinant for FMD, at least under the assumption of classical Mendelian inheritance.

In the same study, the authors selected some genes harbouring rare coding variants without intra-familial segregation, and looked for the frequency of variants in these genes in 249 unrelated FMD patients, also ascertained from the Rare Vascular Diseases Referral Centre, HEGP and 689 controls from the SUpplémentation en VItamines et Minéraux AntioXydants (SU.VI.MAX) study, a national sample of healthy volunteers. They applied a gene-based collapsing analysis named Optimal Sequence Kernel Association Test (SKAT-O), which is recommended for measuring association of multiple rare variants in a given gene instead of testing each individual variant separately, thereby providing more power [43]. Despite overall negative findings, this study unravelled nominally significant association between multifocal FMD and myosin light chain kinase (*MYLK*; previously involved in thoracic aortic aneurysms), dynein cytoplasmic heavy chain 1 gene (*DYNC2H1*), obscurin (*OBSCN*; a sarcomeric protein) and *RNF213*, previously associated with Moyamoya disease (*p* = 0.01) [43]. While these findings need replication, they highlight the importance of accurate phenotyping and classification, to distinguish multifocal FMD from focal FMD, which may have different pathophysiologies, and/or to avoid including phenocopies/FMD mimics.

#### 5.2.2. Potential Genetic Link between the Grange Syndrome and FMD

Recently, Guo and co-workers [44] conducted a whole-exome-sequencing analysis on DNA from two affected siblings of the first reported family affected by Grange syndrome. This is a unique autosomal recessive syndrome, characterized by arterial stenosis that is similar to focal FMD in angiographic appearance and distribution, congenital cardiac defects, brachydactyly, syndactyly, bone fragility and learning disabilities [45]. Using very stringent filtering criteria, and by retaining only loss-of-function mutations, whole exome sequencing led to the identification of two compound heterozygous loss-of-function mutations in the YY1 associated protein 1 gene (*YY1AP1*) in the two siblings [44]. Sanger sequencing confirmed the presence of both *YY1AP1* mutations in the third affected sibling. Interestingly, their mother, who harboured one of the variants, had a stenosis of the proximal renal artery without other syndromic manifestations. Finally, three other unrelated patients with the Grange syndrome were found to harbour distinct *YY1AP1* mutations at the homozygous state.

The *YY1AP1* gene encodes a 88 kDa protein that is part of the INO80 chromatin-remodelling complex, and may play a role in transcriptional regulation and cell cycle control. The authors showed that *YY1AP1* expression is induced during smooth muscle cell differentiation in vitro and that its knockdown prevents the expression of smooth muscle markers and the differentiation of smooth muscle cells. *YY1AP1* loss-of-function mutations may thus lead to an increased presence of proliferating smooth muscle cell precursors and reduced amounts of differentiated quiescent, contractile smooth muscle cells. In view of these elements, the authors hypothesized that heterozygous *YY1AP1* variants may be a rare predisposing allele of FMD [44]. While analysis of whole-exome-sequencing data of 282 patients with FMD and 286 matched controls failed to show an increased burden of *YYAP1* variants in FMD cases vs. controls, a single heterozygous *YY1AP1* frameshift mutation was identified in one out of the 282 cases—a female patient with involvement of both renal and carotid arteries—and none in the controls subjects [44]. Further genetic studies are required to determine the possible contribution of *YYAP1* variants in non-syndromic FMD.

### 5.3. Non-Hypothesis Driven Genetic Association Study

After the limited success of whole-exome-sequencing in related FMD patients and poor yield of screening for rare variants [31,44], alternative hypotheses needed to be explored. Using a genetic association study in 1154 patients with (mostly renal artery) FMD and 3895 controls of European ancestry, Kiando and co-workers reported the first consistent and replicated association. They found that allele A of a genetic variant (rs9349379) of the phosphatase and actin regulator 1 gene (*PHACTR1*), highly prevalent in the general population (~60%), was associated with a 40% increase in relative risk of FMD (OR = 1.39, *p* < 7.36 × 10^−10^) [46]. Moreover, in 2458 healthy volunteers, the authors documented an association between the *PHACTR1* at-risk allele and increased carotid intima-media thickness (IMT) (*p* < 1.65 × 10^−4^) and wall-to-lumen ratio (*p* = 0.002) [46]. Furthermore, while expression of *PHACTR1* did not differ in cultured human fibroblasts from 51 patients with FMD compared to 39 controls, stratification by rs9349379 genotype indicated increased expression in subjects with the at-risk allele, rs9349379[A] (*p* < 0.003) [46]. Finally, *zPhactr1* knockdown was associated with a mild (~8%) but significant (*p* < 0.05) vessel dilatation in zebrafish [46].

**Figure 2:**
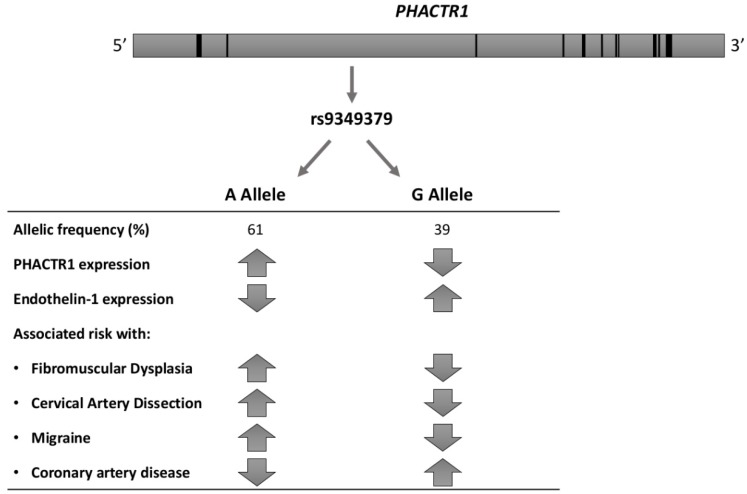
Association of rs9349379 locus with PHACTR1 and Endothelin-1 expression, cardio- and cerebrovascular diseases [46,51].

Already in 2015, the Cervical Artery Dissections and Ischemic Stroke Patients (CADISP) study, a consortium dedicated to the genetics of cervical artery dissection (CeAD), had identified *PHACTR1* as a risk locus for stroke in young adults. In particular, the rs9349379[A] allele was associated with a higher CeAD risk (OR = 1.33, *p* = 4.46 × 10^−10^) [47]. Furthermore, in a meta-analysis of 29 genome-wide association studies (GWAS), including a total of 23285 individuals with migraine and 95,425 population-matched controls, rs9349379[A] was associated with an increased risk of migraine without aura (*p* < 2.81 × 10^−10^) [48], clinical presentation shared by FMD and CeAD. These findings are of interest in view of the overlap between FMD, carotid artery dissection and migraine. Indeed, in a sample of 732 patients with CeAD, the prevalence of documented FMD was 5.6%, with a significant difference between patients with multiple and single CeAD (15% vs. 3.8%, *p* < 0.0001) [49]. Furthermore, headaches are a common symptom in patients with FMD (60% of patients in the US registry, with classical migraine-type headaches reported in 32%) [3]. More intriguingly, in a meta-analysis of four large genome-wide association studies including 15420 individuals with coronary artery disease (CAD) and 15,062 controls, the same allele, rs9349379[A], was associated with a decreased risk of CAD (*p* = 5.8 × 10^−19^) [50]. The associations of the rs9349379 locus with cardio- and cerebrovascular diseases are summarized in Figure 2.

### 5.4. Insights and Limitations from Functional Genomics at PHACTR1 Locus

Although rs9349379 is seemingly positioned in a *PHACTR1* intron, it is actually located about 54 kbp upstream of *PHACTR1* transcription start site used in endothelial and smooth muscle cells of the arteries, and about 265 kbp upstream of another *PHACTR1* transcription start site used in macrophages [52]. The homozygous A allele at rs9349379 was associated with higher *PHACTR1* expression in skin fibroblasts and macrophages in healthy donors [46,53] (Figure 2). Publicly available datasets show an enrichment of histone acetylation marks (H3K27ac) close to rs9349379 in arteries, but not in other analysed tissues, suggesting that the region may serve as a tissue-specific enhancer [51]. The rs9349379[G] allele is predicted to disrupt a bona fide binding site for myocyte enhancer factor 2 (*MEF2*) transcription factors, and binding of MEF2s to this site was indeed shown in vitro [54]. However, knockdown of either *MEF2A* or *MEF2C* in human umbilical vein endothelial cells did not result in a decrease of *PHACTR1* expression.

A recent study used induced pluripotent stem cells and CRISPR-Cas9 modification to study the effect of homozygous rs9349379[A] or [G] on gene expression [51]. Modified cells were differentiated into vascular endothelial or vascular smooth muscle cell lineages, and gene expression was analysed using microarrays and qPCR. The results suggested that the rs9349379[G] genotype increases the expression of endothelin-1 (ET-1), whose precursor is encoded by the *EDN1* gene, located about 600 kbp upstream of rs9349379. This observation was corroborated by the finding of higher levels of Big ET-1, a precursor of endothelin-1, in the plasma of healthy subjects harbouring G allele, at least at the homozygous state. ET-1 is a potent vasoconstrictor, which acts on smooth muscle cells to favour their contraction, proliferation and migration. It also plays a role in vascular relaxation through endothelial cells [36]. Accordingly, endothelin-1 imbalance may play a role in the association of rs9349379 to various cardiovascular diseases, although further exploration of the mechanisms specifically involved in FMD is still required.

## 6. Ongoing Studies

Current efforts to improve diagnostics of FMD aim to identify screening methods that would have a high sensitivity and specificity for FMD, and be easy to use, cost-effective, and available in the clinical practice. Preliminary analysis of the very high-Frequency Ultrasonography for arterial phenotyping in patients with Cervico-cerebral artery dissection, Hypertension, Spontaneous coronary artery dissection and fibromuscular dysplasIA (FUCHSIA) study showed an eutrophic remodelling of the radial artery wall in 11 hypertensive female FMD patients with a peculiar “blurred” pattern [41]. Similar images have also been identified in 5 female SCAD patients [55]. New information is also expected from the Pathophysiological Mechanisms of Fibromuscular Dysplasia (MeDyA) study (ClinicalTrials.gov Identifier: NCT01935752), a pathophysiology study designed to assess endothelial function in patients with FMD compared to hypertensive and healthy controls, and to identify possible plasmatic biomarkers (circulating microparticles, circulating microRNAs, endothelial proteins), and vascular echo-tracking abnormalities related to the FMD.

From a genetic and pathophysiologic point of view, the Defining the Basis of Fibromuscular Dysplasia (DEFINE) study (ClinicalTrials.gov Identifier: NCT01967511) is in the process of recruitment. The researcher’s aim is to establish functional, molecular and genetic profiles of fibroblasts from FMD, SCAD and CeAD patients as compared to matched control subjects, in order to dissect the core biologic mechanisms underlying these disorders. Such data should allow to identify similarities and differences in pathophysiology, which could help stratify patients by using biological markers.

## 7. Future Research Directions and Clinical Perspectives

In view of the relatively modest association of rs9349379[A] with FMD [46], compatible with polygenic inheritance, and the poor yield of screening for mutations in genes involved in CTD [36,37,38], genetic testing has currently no place in the diagnosis and management of the disease outside a research context [1]. Still, progressive dissection of the genetic basis of FMD paves the way for a better understanding of the disease, which may hopefully lead to identification of accurate prognostic biomarkers and novel preventive and therapeutic options.

The association of FMD with the intronic variant in *PHACTR1* needs to be replicated in large and independent cohorts of patients with more balanced representation of renal and cervico-cephalic FMD, and tested in related conditions, such as SCAD. Whether this association is restricted to multifocal FMD or is also present in patients with focal FMD, and whether it is modulated by gender, ethnic background, tobacco exposure and/or other environmental factors remains to be demonstrated. More studies are also needed to better understand the dichotomic effect of the *PHACTR1* locus on FMD and related vascular diseases on one side, and on atherosclerotic coronary artery disease on the other side. Larger cohorts obtained through international collaborations should also facilitate identification of other susceptibility loci associated with FMD. Unveiling of genetic factors underlying FMD and their interactions with environmental factors may allow (i) to propose a more accurate classification of the disease; (ii) to identify subsets of patients with FMD associated with a higher risk of progression/complications; and (iii) to ascertain or not the dysplastic nature of arterial aneurysms and/or dissections occurring in patients without typical string-of-beads or focal FMD stenosis. Such advances in our understanding of the genetic basis of FMD are critically dependent on ongoing efforts to develop large scale registries including patients with FMD and related diseases, such as the US and European/International registries [3,39].

## Figures and Tables

**Table 1:** Differential diagnosis of focal Fibromuscular Dysplasia (FMD) in a nutshell (modified from [1]).

Focal atherosclerotic lesions
Inflammatory arterial diseases: Takayasu arteritis; Giant cell arteritis.
Arterial diseases of monogenic origin: Type 1 neurofibromatosis; Alagille syndrome; Williams syndrome.

## References

[1] Persu A., Giavarini A., Touzé E., Januszewicz A., Sapoval M., Azizi M., Barral X., Jeunemaitre X., Morganti A., Plouin P.F. (2014). European consensus on the diagnosis and management of fibromuscular dysplasia. J. Hypertens..

[2] Sharma A.M., Kline B. (2014). The United States registry for fibromuscular dysplasia: New findings and breaking myths. Tech. Vasc. Interv. Radiol..

[3] Olin J.W., Froehlich J., Gu X., Bacharach J.M., Eagle K., Gray B.H., Jaff M.R., Kim E.S., Mace P., Matsumoto A.H. (2012). The United States registry for fibromuscular dysplasia: Results in the first 447 patients. Circulation.

[4] Plouin P.-F., Baguet J.-P., Thony F., Ormezzano O., Azarine A., Silhol F., Oppenheim C., Bouhanick B., Boyer L., Persu A. (2017). High prevalence of multiple arterial bed lesions in patients with fibromuscular dysplasia: The ARCADIA registry (Assessment of Renal and Cervical Artery Dysplasia). Hypertension.

[5] Olin J.W., Gornik H.L., Bacharach J.M., Biller J., Fine L.J., Gray B.H., Gray W.A., Gupta R., Hamburg N.M., Katzen B.T. (2014). Fibromuscular dysplasia: State of the science and critical unanswered questions: A scientific statement from the American Heart Association. Circulation.

[6] Cragg A.H., Smith T.P., Thompson B.H., Maroney T.P., Stanson A.W., Shaw G.T., Hunter D.W., Cochran S.T. (1989). Incidental fibromuscular dysplasia in potential renal donors: Long-term clinical follow-up. Radiology.

[7] Blondin D., Lanzman R., Schellhammer F., Oels M., Grotemeyer D., Baldus S.E., Rump L.C., Sandmann W., Voiculescu A. (2010). Fibromuscular dysplasia in living renal donors: Still a challenge to computed tomographic angiography. Eur. J. Radiol..

[8] Saw J., Mancini G.B.J., Humphries K.H. (2016). Contemporary review on spontaneous coronary artery dissection. J. Am. Coll. Cardiol..

[9] Kirton A., Crone M., Benseler S., Mineyko A., Armstrong D., Wade A., Sebire G., Crous-Tsanaclis A.-M., deVeber G. (2013). Fibromuscular dysplasia and childhood stroke. Brain J. Neurol..

[10] Savard S., Steichen O., Azarine A., Azizi M., Jeunemaitre X., Plouin P.-F. (2012). Association between 2 angiographic subtypes of renal artery fibromuscular dysplasia and clinical characteristics. Circulation.

[11] Touzé E., Oppenheim C., Trystram D., Nokam G., Pasquini M., Alamowitch S., Hervé D., Garnier P., Mousseaux E., Plouin P.-F. (2010). Fibromuscular dysplasia of cervical and intracranial arteries. Int. J. Stroke Off. J. Int. Stroke Soc..

[12] Stanley J.C., Gewertz B.L., Bove E.L., Sottiurai V., Fry W.J. (1975). Arterial fibrodysplasia. Histopathologic character and current etiologic concepts. Arch. Surg..

[13] Ross R., Klebanoff S.J. (1971). The smooth muscle cell. I. In vivo synthesis of connective tissue proteins. J. Cell Biol..

[14] Lüscher T.F., Lie J.T., Stanson A.W., Houser O.W., Hollier L.H., Sheps S.G. (1987). Arterial fibromuscular dysplasia. Mayo Clin. Proc..

[15] Sang C.N., Whelton P.K., Hamper U.M., Connolly M., Kadir S., White R.I., Sanders R., Liang K.-Y., Bias W. (1989). Etiologic factors in renovascular fibromuscular dysplasia. A case-control study. Hypertension.

[16] Leung D.Y., Glagov S., Mathews M.B. (1976). Cyclic stretching stimulates synthesis of matrix components by arterial smooth muscle cells in vitro. Science.

[17] Kaufman J.J., Maxwell M.H. (1963). Upright aortography in the study of nephroptosis, stenotic lesions of the renal artery, and hypertension. Surgery.

[18] Miller D.J., Marin H., Aho T., Schultz L., Katramados A., Mitsias P. (2014). Fibromuscular dysplasia unraveled: The pulsation-induced microtrauma and reactive hyperplasia theory. Med. Hypotheses..

[19] Sottiurai V., Fry W.J., Stanley J.C. (1978). Ultrastructural characteristics of experimental arterial medial fibroplasia induced by vasa vasorum occlusion. J. Surg. Res..

[20] Fievez M.L. (1984). Fibromuscular dysplasia of arteries: A spastic phenomenon?. Med. Hypotheses..

[21] Mackay A., Brown J.J., Cumming A.M., Isles C., Lever A.F., Robertson J.I. (1979). Smoking and renal artery stenosis. Br. Med. J..

[22] Nicholson J.P., Teichman S.L., Alderman M.H., Sos T.A., Pickering T.G., Laragh J.H. (1983). Cigarette smoking and renovascular hypertension. Lancet.

[23] Boutouyrie P., Gimenez-Roqueplo A.-P., Fine E., Laloux B., Fiquet-Kempf B., Plouin P.-F., Jeunemaitre X., Laurent S. (2003). Evidence for carotid and radial artery wall subclinical lesions in renal fibromuscular dysplasia. J. Hypertens..

[24] Savard S., Azarine A., Jeunemaitre X., Azizi M., Plouin P.-F., Steichen O. (2013). Association of smoking with phenotype at diagnosis and vascular interventions in patients with renal artery fibromuscular dysplasia. Hypertension.

[25] O’Connor S., Gornik H.L., Froehlich J.B., Gu X., Gray B.H., Mace P.D., Sharma A., Olin J.W., Kim E.S.H. (2016). Smoking and Adverse Outcomes in Fibromuscular Dysplasia: U.S. Registry Report. J. Am. Coll. Cardiol..

[26] Halpern M.M., Sanford H.S., Viamonte M. (1965). Renal-artery abnormalities in three hypertensive sisters. Probable familial fibromuscular hyperplasia. JAMA.

[27] Hansen J., Holten C., Thorborg J.V. (1965). Hypertension in two sisters caused by so-called fibromuscular hyperplasia of the renal arteries. Acta Med. Scand..

[28] Foster J.H., Oates J.A., Rhamy R.K., Klatte E.C., Burko H.C., Michelakis A.M. (1969). Hypertension and fibromuscular dysplasia of the renal arteries. Surgery.

[29] Plagnol P., Gillet J.M., Cambuzat J.M., Broussin J. (1975). Familial reno-vascular hypertension. J. Radiol. Electrol. Med. Nucl..

[30] Morimoto S., Kuroda M., Uchida K., Funatsu T., Yamamoto I., Hashiba T., Kametani T., Takeda R., Matsubara F. (1976). Occurrence of renovascular hypertension in two sisters. Nephron.

[31] Kiando S.R., Barlassina C., Cusi D., Galan P., Lathrop M., Plouin P.-F., Jeunemaitre X., Bouatia-Naji N. (2015). Exome sequencing in seven families and gene-based association studies indicate genetic heterogeneity and suggest possible candidates for fibromuscular dysplasia. J. Hypertens..

[32] Pannier-Moreau I., Grimbert P., Fiquet-Kempf B., Vuagnat A., Jeunemaitre X., Corvol P., Plouin P.-F. (1997). Possible familial origin of multifocal renal artery fibromuscular dysplasia. J. Hypertens..

[33] Green R., Gu X., Kline-Rogers E., Froehlich J., Mace P., Gray B., Katzen B., Olin J., Gornik H.L., Cahill A.M. (2016). Differences between the pediatric and adult presentation of fibromuscular dysplasia: Results from the US Registry. Pediatr. Nephrol..

[34] Rushton A.R. (1980). The genetics of fibromuscular dysplasia. Arch. Intern. Med..

[35] Perdu J., Boutouyrie P., Bourgain C., Stern N., Laloux B., Bozec E., Azizi M., Bonaiti-Pellié C., Plouin P.-F., Laurent S. (2007). Inheritance of arterial lesions in renal fibromuscular dysplasia. J. Hum. Hypertens..

[36] Ganesh S.K., Morissette R., Xu Z., Schoenhoff F., Griswold B.F., Yang J., Tong L., Yang M.-L., Hunker K., Sloper L. (2014). Clinical and biochemical profiles suggest fibromuscular dysplasia is a systemic disease with altered TGF-β expression and connective tissue features. FASEB J..

[37] O’Connor S., Kim E.S., Brinza E., Moran R., Fendrikova-Mahlay N., Wolski K., Gornik H.L. (2015). Systemic connective tissue features in women with fibromuscular dysplasia. Vasc. Med. Lond. Engl..

[38] Poloskey S.L., Kim E.S., Sanghani R., Al-Quthami A.H., Arscott P., Moran R., Rigelsky C.M., Gornik H.L. (2012). Low yield of genetic testing for known vascular connective tissue disorders in patients with fibromuscular dysplasia. Vasc. Med. Lond Engl..

[39] Persu A., van der Niepen P., Touzé E., Gevaert S., Berra E., Mace P., Plouin P.-F., Jeunemaitre X. (2016). Revisiting Fibromuscular Dysplasia: Rationale of the European Fibromuscular Dysplasia Initiative. Hypertension.

[40] Marais L., Boutouyrie P., Khettab H., Boulanger C., Lorthioir A., Franck M., Niarra R., Renard J., Chambon Y., Jeunemaitre X. (2016). Structural and functional arterial abnormalities in fibromuscular dysplasia are in the continuum of hypertension: An imaging and biomechanical study. Artery Res..

[41] Bruno R.M., Lascio N.D., Guarino D., Vitali S., Rossi P., Caramella D., Taddei S., Ghiadoni L., Faita F. (2017). Abstract P509: Identification of radial vascular wall abnormalities by very-high frequency ultrasound in patients with fibromuscular dysplasia: The fuchsia study. Hypertension.

[42] Marks S.D., Gullett A.M., Brennan E., Tullus K., Jaureguiberry G., Klootwijk E., Stanescu H.C., Kleta R., Woolf A.S. (2011). Renal FMD may not confer a familial hypertensive risk nor is it caused by *ACTA2* mutations. Pediatr. Nephrol..

[43] Lee S., Wu M.C., Lin X. (2012). Optimal tests for rare variant effects in sequencing association studies. Biostatistics.

[44] Guo D.-C., Duan X.-Y., Regalado E.S., Mellor-Crummey L., Kwartler C.S., Kim D., Lieberman K., de Vries B.B.A., Pfundt R., Schinzel A. (2017). Loss-of-function mutations in *YY1AP1* lead to grange syndrome and a fibromuscular dysplasia-like vascular disease. Am. J. Hum. Genet..

[45] Grange D.K., Balfour I.C., Chen S.C., Wood E.G. (1998). Familial syndrome of progressive arterial occlusive disease consistent with fibromuscular dysplasia, hypertension, congenital cardiac defects, bone fragility, brachysyndactyly, and learning disabilities. Am. J. Med. Genet..

[46] Kiando S.R., Tucker N.R., Castro-Vega L.-J., Katz A., D’Escamard V., Tréard C., Fraher D., Albuisson J., Kadian-Dodov D., Ye Z. (2016). *PHACTR1* is a genetic susceptibility locus for fibromuscular dysplasia supporting its complex genetic pattern of inheritance. PLoS Genet..

[47] Debette S., Kamatani Y., Metso T.M., Kloss M., Chauhan G., Engelter S.T., Pezzini A., Thijs V., Markus H.S., Dichgans M. (2015). Common variation in *PHACTR1* is associated with susceptibility to cervical artery dissection. Nat. Genet..

[48] Anttila V., Winsvold B.S., Gormley P., Kurth T., Bettella F., McMahon G., Kallela M., Malik R., de Vries B., Terwindt G. (2013). Genome-wide meta-analysis identifies new susceptibility loci for migraine. Nat. Genet..

[49] Béjot Y., Aboa-Eboulé C., Debette S., Pezzini A., Tatlisumak T., Engelter S., Grond-Ginsbach G., Touzé E., Sessa M., Metso T. (2014). Characteristics and outcomes of patients with multiple cervical artery dissection. Stroke.

[50] Coronary Artery Disease (C4D) Genetics Consortium (2011). A genome-wide association study in Europeans and South Asians identifies five new loci for coronary artery disease. Nat. Genet..

[51] Gupta R.M., Hadaya J., Trehan A., Zekavat S.M., Roselli C., Klarin D., Emdin C.A., Hilvering C.R.E., Bianchi V., Mueller C. (2017). A Genetic variant associated with five vascular diseases is a distal regulator of endothelin-1 gene expression. Cell.

[52] Perdu J., Gimenez-Roqueplo A.-P., Boutouyrie P., Beaujour S., Laloux B., Nau V., Fiquet-Kempf B., Emmerich J., Tichet J., Plouin P.-F. (2006). Alpha1-antitrypsin gene polymorphisms are not associated with renal arterial fibromuscular dysplasia. J. Hypertens..

[53] Reschen M.E., Lin D., Chalisey A., Soilleux E.J., O’Callaghan C.A. (2016). Genetic and environmental risk factors for atherosclerosis regulate transcription of phosphatase and actin regulating gene *PHACTR1*. Atherosclerosis.

[54] Beaudoin M., Gupta R.M., Won H.-H., Lo K.S., Do R., Henderson C.A., Lavoie-St-Amour C., Langlois S., Rivas D., Lehoux S. (2015). Myocardial infarction-associated SNP at 6p24 interferes with *MEF2* binding and associates with *PHACTR1* expression levels in human coronary arteries. Arterioscler. Thromb. Vasc. Biol..

[55] Bruno R.M., di Lascio N., Al Hussaini A., Guarino D., Vitali S., Rossi P., Caramella D., Cortese B., Faita F., Taddei S. (2017). Disarray and remodeling of the radial artery in women with spontaneous coronary artery dissection: The FUCHSIA study. Artery Res..

